# Modular Protein Expression Toolbox (MoPET), a standardized assembly system for defined expression constructs and expression optimization libraries

**DOI:** 10.1371/journal.pone.0176314

**Published:** 2017-05-17

**Authors:** Ernst Weber, Jörg Birkenfeld, Jürgen Franz, Uwe Gritzan, Lars Linden, Mark Trautwein

**Affiliations:** 1 Antibody Lead Discovery, Biologics Research, Bayer AG, Cologne, Germany; 2 Cell and Protein Sciences, Biologics Research, Bayer AG, Wuppertal, Germany; 3 Protein Engineering and Assays, Biologics Research, Bayer AG, Cologne, Germany; New England Biolabs Inc, UNITED STATES

## Abstract

The design and generation of an optimal expression construct is the first and essential step in in the characterization of a protein of interest. Besides evaluation and optimization of process parameters (e.g. selection of the best expression host or cell line and optimal induction conditions and time points), the design of the expression construct itself has a major impact. However, the path to this final expression construct is often not straight forward and includes multiple learning cycles accompanied by design variations and retesting of construct variants, since multiple, functional DNA sequences of the expression vector backbone, either coding or non-coding, can have a major impact on expression yields. To streamline the generation of defined expression constructs of otherwise difficult to express proteins, the Modular Protein Expression Toolbox (MoPET) has been developed. This cloning platform allows highly efficient DNA assembly of pre-defined, standardized functional DNA modules with a minimal cloning burden. Combining these features with a standardized cloning strategy facilitates the identification of optimized DNA expression constructs in shorter time. The MoPET system currently consists of 53 defined DNA modules divided into eight functional classes and can be flexibly expanded. However, already with the initial set of modules, 792,000 different constructs can be rationally designed and assembled. Furthermore, this starting set was used to generate small and mid-sized combinatorial expression optimization libraries. Applying this screening approach, variants with up to 60-fold expression improvement have been identified by MoPET variant library screening.

## Introduction

Production of soluble and active recombinant protein in sufficient amounts is central for structural, functional and biochemical protein characterization. However, the path to an optimal expression construct for a gene of interest (GOI) is often a tedious trial and error process. Multiple variables can influence expression and function. In particular choice of the expression host, expression conditions, expression vector backbone, promoters, terminators, signal peptides, different N- and/or C—terminal protein tags, linkers and combinations thereof have to be explored and tested in order to achieve optimal protein expression constructs tailored for an application of interest. Modifications and adaptations of a given expression construct, like exchanging a protein tag or replacing the promoter, generally require individual cloning strategies which is time-consuming and resource intensive.

Creating large numbers of DNA construct variants with defined functions can be substantially streamlined by applying engineering principles to the field of molecular biology. In particular standardization of basic parts and the assembly process itself have revolutionized well established engineering disciplines.

A first step to standardize DNA expression construct generation (= assembly process) was the development of universal cloning strategies like Flexi^®^ cloning (Promega), Creator^™^ DNA cloning system (Clontech) or Gateway^®^ (Life Technologies). In all systems the GOI is cloned by either defined rare cutting restriction endonucleases or recombinases into vector backbones adapted to the intended cloning approach. To match the requirements of a defined cloning system, recombination or endonuclease recognition sites have to be added to the GOIs, consequently, translating into additional and thus unwanted amino acids which can have an unpredictable effect on the protein of interest (POI).

Variability of the resulting expression constructs is then achieved by cloning the GOI into alternative backbones equipped with predefined genetic elements. This has led to versatile plasmid collections for each of the described cloning systems. However, this vector sets will always cover only a fraction of all combinatorial possibilities of the genetic elements. A preferred combination, e.g. backbone with a desired tag and promoter may be missing, and additional cloning efforts are needed to generate this defined vector.

Free combination of promoters, GOIs and tags in multiple arrangements and generation of combinatorial expression construct libraries, requires that the genetic elements themselves have to be standardized and be by this process transformed into biological parts. In recent years universal cloning strategies have been developed for the assembly of standardized biological parts. Beside the BioBricks^™^ standard, where an assembly protocol based on standard type IIR restriction endonucleases is used, two systems using type IIS enzyme based Golden Gate cloning technologies, GoldenBraid and Modular Cloning (MoClo) have been described [1–4]. Golden Gate cloning permits directional and seamless assembly of multiple DNA fragments in a one-tube one-step reaction by the concurrent use of type IIS restriction endonucleases and DNA ligase. The method proved to be highly robust and efficient in DNA shuffling applications and for assembly of highly repetitive DNA sequences [5–11].

MoClo and GoldenBraid represent both standardized systems which allow defined generation of complex multi gene constructs, with sizes of more than 100kb, which are of special interest in the field of metabolic pathway engineering in plants when multiple genes have to be assembled [12]. Based on the same methodology further cloning systems focusing on certain model organisms like plants, yeast and *E*.*coli* have been developed [13–16].

As an extension we describe here the MoPET platform, a toolbox for standardized assembly of expression constructs and protein expression libraries to optimize protein expression in eukaryotic expression hosts. The system has a strong focus on the special requirements when working in coding regions and is designed to minimize cloning burden like additional amino acids between the functional parts which may influence expression and/or function of the POI. The unique feature of Golden Gate cloning, to require only a single amino acid for the fusion of two functional parts, is the key differentiator to other widely used cloning technologies. Methods like e.g. Gibson assembly [17], SLIC [18] and related technologies reviewed by Casini et al. [19], which are also able to combine multiple DNA fragments with high efficiency, need longer identical sequence stretches between the single parts (15–30 nucleotides). The longer sequences have restrictions when used in modular toolboxes: i) they have the potential to insert larger stretches of unwanted amino acids, ii) highly repetitive sequences like GS-linkers are problematic in efficient assembly, iii) very short functional parts like tags are not possible as independent single entities, and iv) a simple expansion by adding new functional parts may require major adjustments in the cloning system. This becomes even more prominent when multiple functional parts are assembled in a linear fashion together with multiple fusion sites in the coding region. We specified for MoPET eight functional module types, covering the main biological components of expression constructs: promoters, signal peptides, N-terminal and C- terminal tags and linkers and plasmid backbones with designed fusion sites connecting the module types without adding undesired amino acids to the final constructs. Up to eight different module types can be assembled in a single one-pot reaction to form defined constructs with high efficiency. Moreover, the system allows generation of construct libraries for expression scouting when multiple module variants of a single class are combined in one assembly reaction. The system described here consists of a starting set of 53 modules allocated to eight module classes and allows the generation of 792,000 different constructs, and can be easily expanded to meet the researcher’s needs. We show here that with generation of even medium sized expression optimization libraries expression levels of recombinant proteins produced in mammalian cells can be increased by up to 60-fold.

## Material and methods

### Molecular biology reagents

Restriction enzymes used in this study were purchased from New England Biolabs (Ipswich, MA) and Fermentas (Burlington, Canada). T4 DNA ligase was purchased from Promega (Fitchburg, WI). Plasmid DNA preparations were made by using the QIAprep Spin miniprep Kit (Qiagen, Hilden, Germany) following the manufacturer’s protocol. Plasmid DNA concentration was measured using a Nano Drop^®^ Spectrophotometer ND-1000 (Peqlab, Erlangen, Germany).

### Vector construction

Level 0 functional modules of the SP, N-tag, C-tag, N-Link, and C-Link types were designed and ordered at Life Technologies^™^ (Carlsbad, CA) as human codon optimized DNA constructs in plasmids conferring kanamycin resistance and lacking BsaI restriction sites. Level 0 promoter modules were domesticated when necessary, i.e. the BsaI and BpiI sites were removed via Golden Gate cloning. In brief, PCR Primers were designed with type IIS restriction sites and fusion sites, which altered sequence of the type IIS recognition site present in the original sequence. The PCR fragments were cloned via standard cloning technologies into a pUC derivative conferring kanamycin resistance [20].

In the domestication process of the level 0 destination plasmids BsaI, BpiI and SapI were removed by PCR and assembly of the respective PCR fragments via Golden Gate cloning. In this process also all biological functions which are covered by level 0 modules, like promoters and tags, were removed and a type IIS entry option combined with a lacZ gene was introduced. In brief, the lacZ gene was amplified and incorporated together with the PCR products, generated to domesticate the plasmids, into the Golden Gate assembly reaction [4, 20]. All modules with their coding sequence will be provided as supplementary sequence file (S1 File: Module sequence information). The sequence of the CMV-5 promoter has to be obtained for contractual reasons from the National Research Council Canada.

### Standard Moclo cloning protocol

A one-step—one-pot restriction/ligation was set up with approximately 30 fmol (~100 ng for a 5 kb plasmid) of each plasmid., Promega ligation buffer, 10 U of the respective restriction enzyme (BsaI, BpiI), 10 U high concentrated T4 DNA ligase, in a 20 μl volume. The reaction was incubated for 2 hours at 37°C, 5 min at 50°C and 5 min at 80°C. The mix was added to 50 μl electro competent Top10 cells, incubated for 15–30 min on ice and transformed by electroporation. Clones were analyzed by restriction analysis, colony PCR and optionally by sequencing.

### Expression and quantification of hPTK7

Transient production in suspension HEK293–6E cells was performed as previously described in detail [21, 22]. Briefly, TubeSpin^®^ Bioreaktor 50 (TPP; 35 ml culture volume) or 96 half deep well plates using sandwich covers (Enzyscreen—System Duetz; 200 μl culture volume) have been used for expression. To determine the predictivity of a high throughput transfection system to a later upscale in expression, mini-prep derived DNA was used for DWP expression and midi-prep derived DNA for expression in 35 ml TubeSpins. Five days post-transfection, the cultures were cleared from cells by centrifugation. Expression levels were determined by Protein A chromatography on an Agilent 1200 HPLC system (POROS A/20 2.1 mm D x 30 mm L; Applied Biosystems). The resulting peak was detected at OD 280 nm, integrated and quantified using a human IgG1 as reference for calibration.

## Results

### Conceptual design of the modular protein expression toolbox (MoPET)

The MoPET assembly strategy described here uses the Golden Gate cloning method, which is based on the special ability of type IIS enzymes to cleave outside their recognition site in a defined distance independently of the target sequence. When these recognition sites are placed to the far 5’ and 3’ end of any DNA fragment in inverse orientation they are removed by the restriction enzyme in the cleavage process. Two DNA fragments flanked by compatible sequence overhangs resulting from type IIS cleavage, termed fusion sites, can be ligated seamlessly (S1 Fig). Since type IIS sites can be designed to have different fusion site sequences, directional assembly of multiple fragments is feasible. For the assembly reaction, all DNA fragments can be simply provided as plasmid preparations, and are combined with the destination plasmid, T4 DNA ligase and the type IIS restriction enzyme in a single reaction mix, reducing the number of handling steps to an absolute minimum. We have shown earlier that up to 10 DNA fragments can be assembled in a one-pot one-step reaction with 95-100% of colonies containing the expected construct [4, 6].

**Fig 1:**
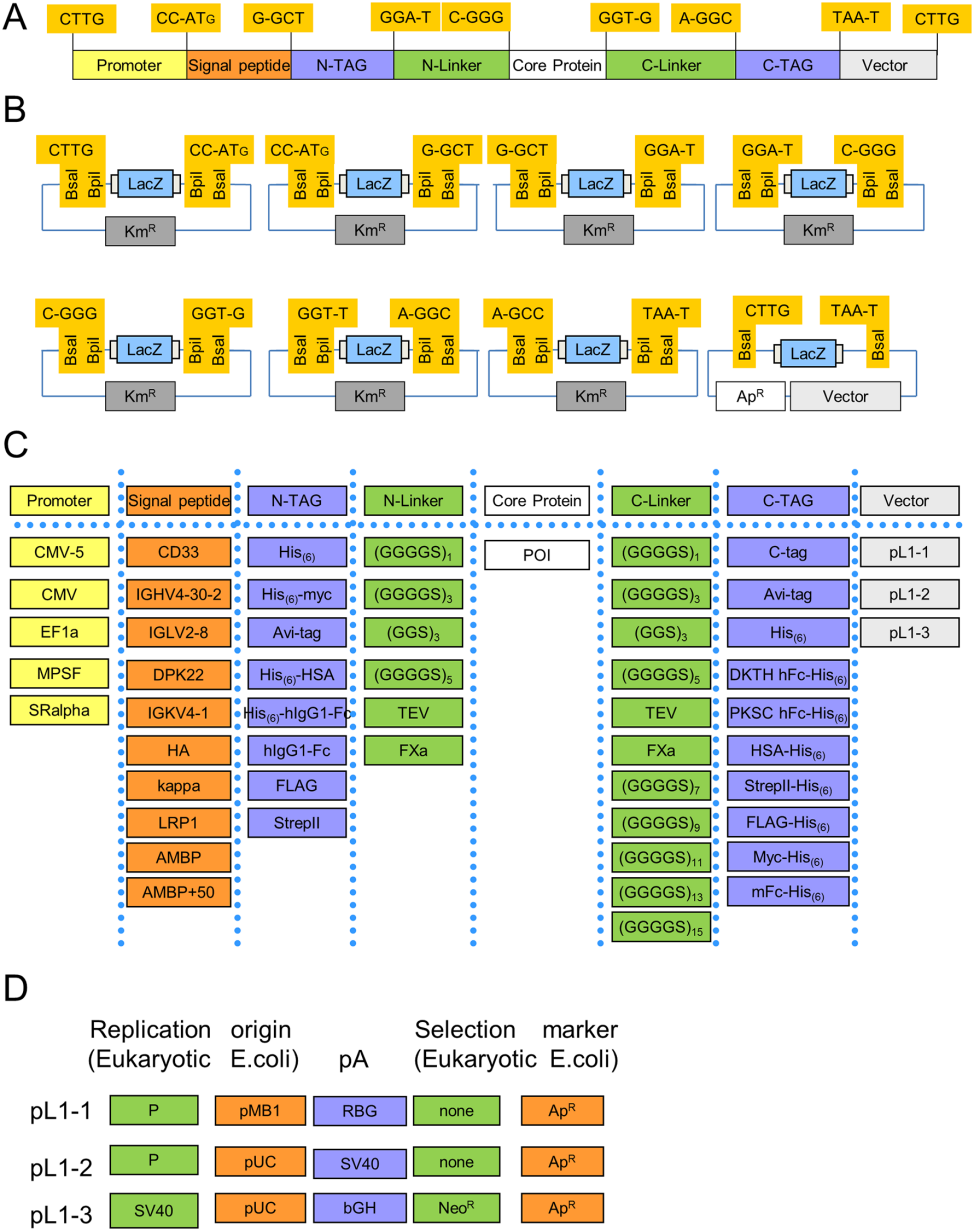
General overview of the MoPET design and implemented functional parts. (A) Modular structure of the MoPET system consisting of the eight basic module types Promoter, Signal Peptide, N-TAG (N-terminal tag), N-Linker (N-terminal linker), Core Protein, C-Linker (C-terminal linker), C-TAG (C-terminal tag) and the vector. Boxes show the fusion sites separating the modules and indicating the reading frame. (B) Layout of level 0 storage plasmids for the respective module positions and the destination backbones. The level 0 storage plasmids confer resistance to kanamycin and allow blue white selection for cloning purposes. Cloning into this plasmid set can be performed via BpiI, with a final BsaI based assembly in the ampicillin resistant level 1 backbones. (C) Compilation of the functional modules compatible with the MoPET system. DKTH-hFc-His (human IgG1 Fc sequence starting with DKTH), PKSC-hFc-His (human IgG1 Fc sequence starting with PKSC), HSA (human serum albumin), mFc (murine Fc sequence), Avi-tag [24]. (D) Overview of the functional features of the three basic backbones. P: OriP [25], pA: poly adenylation site, RBG: rabbit beta-globin [26], SV40 Simian virus 40, bGH: bovine growth hormone [27], Neo^R^: neomycin resistance, Ap^R^: ampicillin resistance, Km^R^: kanamycin resistance.

Based on this assembly technology we set up MoPET to allow the systematic and standardized assembly of expression constructs and expression construct libraries. In a first step we defined eight basic module types which were named in accordance with the MoClo system syntax as level 0 modules [4]. They cover the main variables in expression construct design and optimization, such as promoter (P), signal peptide (SP), N-terminal tag (N-TAG), N-terminal linker (N-Linker), the core of the protein to be expressed (CP), C-terminal linker (C-Linker), and C-terminal tag (C-TAG) (Fig 1A). The plasmid backbone itself is also regarded as a module, providing additional expression level determining functions like origin of replication and the 3’-UTR further. To enable assembly by Golden Gate technology and to assure that all modules of a defined type are interchangeable with each other, we defined specific fusion sites flanking each module type (Fig 1A). Fusion sites overlapping with coding sequences were chosen so as to minimize changes to the encoded proteins. The fusion site between promoter and the start of the protein was chosen to be CCAT. The two cytosine residues represent the last two nucleotides of an optimal Kozak sequence for mammalian expression, which are followed by the two first nucleotides of a start AT**-**G. In order to allow creation of the original N-terminus of any given protein to express, the fusion site between the signal peptide and the N-terminal tag is located in the coding sequence of the signal peptide. As alanine is one of the consensus residues for the signal peptidase and present in many mammalian signal peptides at the COOH terminus, the fusion site here reads G-GCT [23]. Definition of two linker positions (N-Linker and C-Linker) flanking the core protein allowed to design fusion sites that were located inside the linker coding region. As the majority of linker sequences used in literature typically contain glycine, and a single additional glycine in front of the linkers is regarded as minor disturbance of the expression construct, all four glycine codons were used (GGA-T, C-GGG, GGT-G, A-GGC). As fusion site to connect the POI to the backbone, the TAA-T stop codon was chosen. The last remaining fusion site is located in non-translated region upstream of the promoter module and was selected with the only requirements as to be unique and non-palindromic to allow efficient assembly (CTTG).

### Level 0 module selection, generation and domestication of level 1 destination plasmids

In order to allow efficient and convenient cloning of level 0 modules, a set of level 0 destination vectors was created (Fig 1B). For each module type a specific level 0 destination vector was defined for the five standard elements (pL0-P, pL0-SP, pL0-N-TAG, pL0-N-Linker, pL0-CP. pL0-C-Linker and pL0-C-TAG). All level 0 destination vectors are based on a pUC19 backbone derivative that confers kanamycin resistance (Km^R^) and encodes a lacZα fragment for blue/white selection. On both sides of the lacZα fragment two different type IIS recognition sequences—here BsaI and BpiI—are positioned in inverse orientation relative to each other, but creating the identical fusion site [4]. This design allows cloning of the DNA fragment of interest efficiently via BpiI—removing the BpiI recognition sites and lacZα in the process—but provides the possibility to release the cloned fragment with BsaI creating the identical fusion sites it was cloned in. For cloning of level 0 modules, designated sequences are PCR-amplified, adding the respective fusion site and a BpiI recognition site as part of the primers used for amplification, and cloned via a BpiI Golden Gate cloning reaction. Any internal type IIS recognition site for enzymes used in the MoClo system (BsaI, BpiI) can be removed from the cloned fragment during this step by using primers overlapping but containing a single silent nucleotide mismatch in the recognition site [5]. The removal of the type IIS restriction sites used in the system is called domestication. Alternatively modules can also be ordered at gene synthesis providers disallowing BsaI sites, as in this case no subcloning step using BpiI is required and the following cloning steps are BsaI based only. The complete overview of all initial modules being part of the MoPET system is given in Fig 1C.

Level 0 modules will be assembled to complete transcriptional units in level 1 expression plasmids. In contrast to the level 0 module plasmids, all level 1 destination plasmids confer resistance to ampicillin to allow selection pressure towards correctly assembled constructs as all other modules in the Golden Gate reaction are kanamycin resistant. During the domestication process for pL1-destination plasmids, all BsaI recognition sites were removed in a single Golden Gate reaction based on the same principle as described for generation of Level 0 modules. In the same reaction also promoter sequences and Gateway sites used previously for cloning, and all protein coding DNA fragments present in the original sequence of the expression plasmids were removed and replaced by a lacZα fragment flanked by BsaI sites generating the fusion sites CTTG/TAAT, which allow the assembly of seven level 0 modules; starting from the promoter and extending to the C-terminal tag. The level 1 destination backbones provide different 3’UTR sequences and episomal elements, but all confer ampicillin resistance and carry a high copy origin for propagation in *E*. *coli*. An overview of functional elements present in the final expression backbones are listed in Fig 1D.

### Assembly efficiency evaluation of the Golden Gate based MoPET system

**Fig 2:**
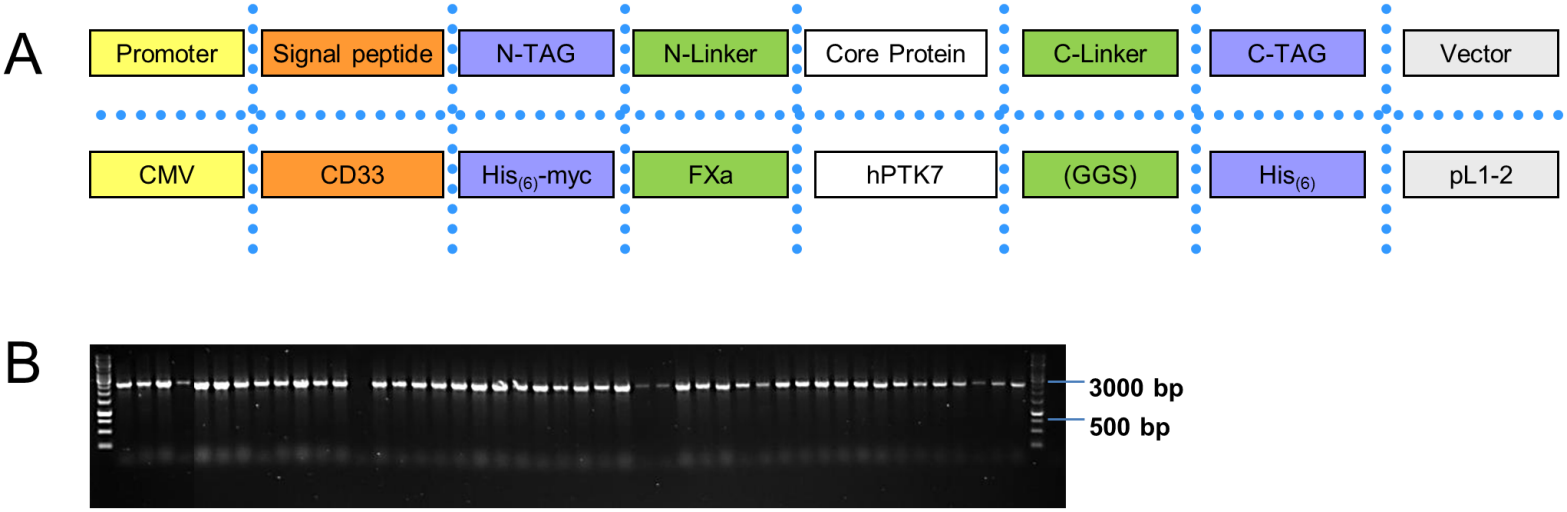
Assembly validation of MoPET. (A) Module composition of the artificial test constructs. Each module was used in an equimolar amount in the assembly reaction (B) Inserts of 47 randomly selected clones where analyzed by colony PCR and visualized on an agarose gel.. M: GeneRuler 1 kb DNA Ladder from ThermoFisher.

In order to determine cloning efficiencies for a construct consisting of all eight functional modules, one of each type was randomly chosen and assembled via a standard Golden Gate assembly protocol (Fig 2A). We had a specific focus on the highly similar fusion sites used for the linker modules, which all include a glycine and in addition added the shortest modules in our initial set to address the efficiency of the assembly of DNA-stretches with only 18 nucleotides. After plating we obtained ~400 white colonies and 1 blue colony. 47 white colonies were picked randomly and a colony PCR with backbone specific primers was performed. 46/47 showed the expected insert size (Fig 2B), however to validate also presence especially of the short FXa (6 aa), His_(6)_ (6 aa), and GGS_(3)_ (9aa) modules, sequences were verified by Sanger sequencing. Based on this experiment we demonstrated the robust and efficient assembly of up to 8 modules, including those only spanning a few amino-acids.

### Using MoPET libraries to optimize expression yield of difficult to express proteins

The standardized assembly strategy implemented in MoPET offers a convenient option to plan multiple defined expression constructs, on the drawing board and assemble them in defined one-pot reactions with a minimal resource investment as each reaction results in more than >95% correct clones.

However, in cases where rational design does not lead to constructs with suitable expression titers, the standardized and modular system also allows to generate expression optimization libraries from the same set of modules applying an identical assembly strategy. In contrast to the standard assembly procedure, where one selected member of each module class is combined, for a library approach module sets for each position can be selected and combined in a single one-pot reaction.

**Fig 3:**
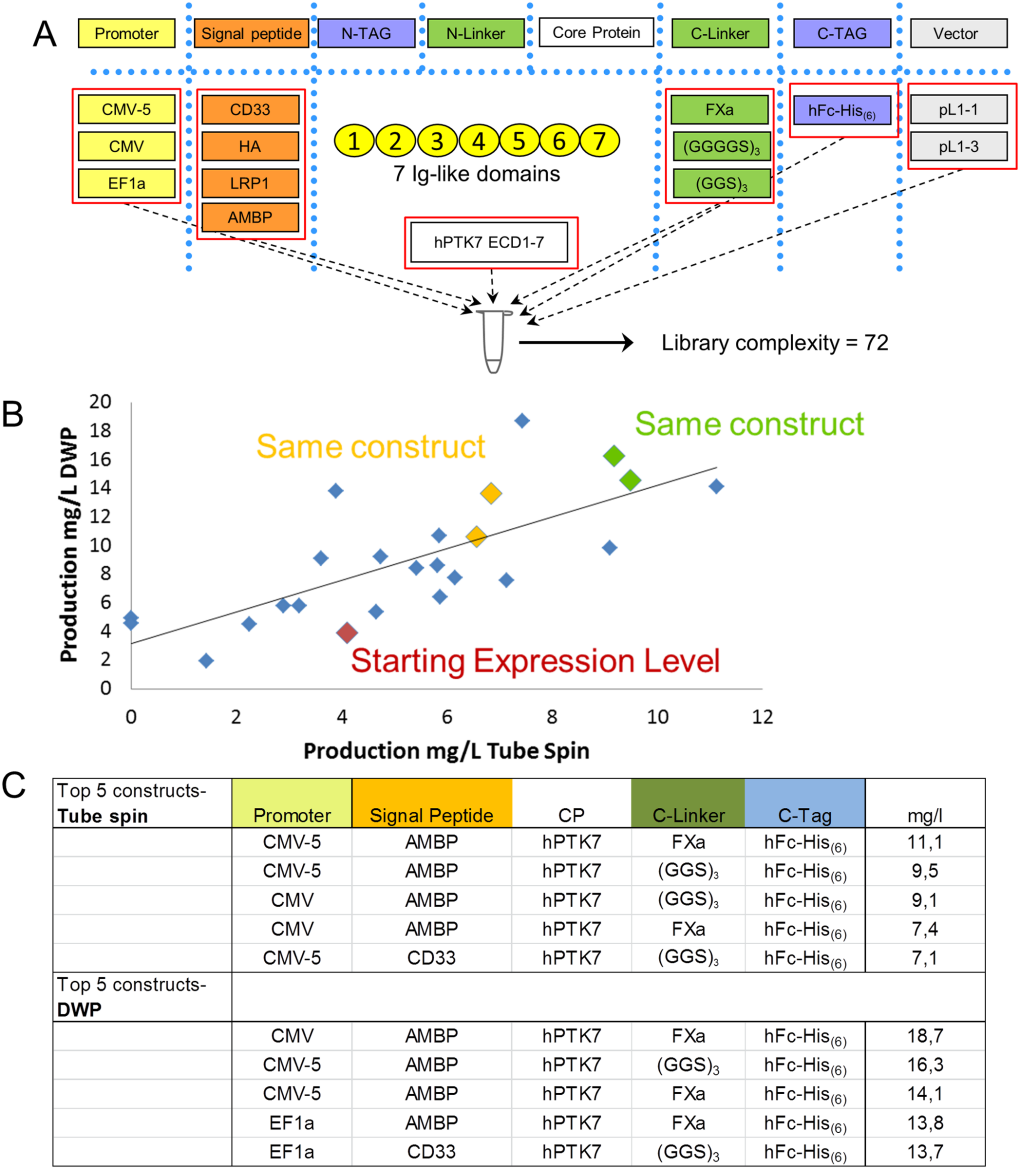
Expression test library of hPTK7-ECD1-7. (A) hPTK7-ECD1-7 expression optimization library design. All listed functional modules were included in a single reaction resulting in a theoretical complexity for the final library of 72. (B) Expression test of 20 unique expression clones in deep well expression (DWP) system (y-axis) or in a tube spin expression system (x-axis). Red diamond represents starting expression level of the original construct. Yellow and green diamonds show the expression level of duplicates of the same construct (yet derived from independent E. coli clones) for 2 cases (Pearson’s Correlation r = 0.7380, R2 = 0.5447, P (two-tailed) <0.0001). (C) Construct design of the Top 5 expression constructs in DWP and Tube spin expression.

As a first proof of concept we selected an established in house expression reference protein: the human tyrosine-protein kinase-like 7 protein (UniProt Identifier: Q13308). The yield for the initial construct design was ~4mg/L (CMV-5 promoter, CD33 signal peptide, hPTK7 ECD1-7 (amino acids 1–680), FXa protease cleavage site and a human IgG1-Fc tag in the expression backbone pL1-1). Based on this initial design a small expression optimization library was designed (Fig 3A). Three promoters, four signal peptides, three C-terminal linkers and two backbone modules were selected, resulting in a library with a theoretical complexity of 72. In the assembly reaction equal concentrations of each module position was used, and for each module position the final concentration was split equally between the modules of the same type. From this library, several clones were randomly selected and sequenced.

We then focused on 20 unique constructs identified in pL1-1 backbone and transfected those in HEK293-6E cells. As controls, we included duplicates of the same construct (yet derived from independent *E*. *coli* clones) for 2 cases and also included one case in both pL1-1 and pL1-3 backbone, and finally the starting construct—in total 24 transfections.

In order to validate the HTS-compatible expression optimization test system, expression titers were analyzed for expression in 96 well deep well plates (DWP) (200 μl) and the well-established TubeSpin system (35 ml) (Fig 3B).

The constructs showed a clear correlation between both expression systems. In addition, identical but independent constructs had matching expression levels (Fig 3B shown in yellow and green). When analyzing successful constructs more closely (Fig 3C), it appeared that the signal peptide had the greatest impact, with AMBP leader being enriched under the top five constructs. Using this very limited library and only expressing 20 unique variants thereof, it was possible to raise the titer by 2–3.5-fold to exceed 10 mg/L.

### Expression scouting and optimization with MoPET

After showing the general feasibility of the approach with a tool compound providing a medium expression level, which could be improved by a factor of 2–3, we designed an entirely artificial protein as a further test candidate. In initial experiments the expression levels for this artificial protein were only detectable by ELISA (~ 100ng/ml). Since no successful expression conditions were reported for this artificial protein entity, MoPET was used to guide us, which elements in which combination may result in the best expressing protein variant. With this initial expression scouting and optimization were combined in a single experiment.

**Fig 4:**
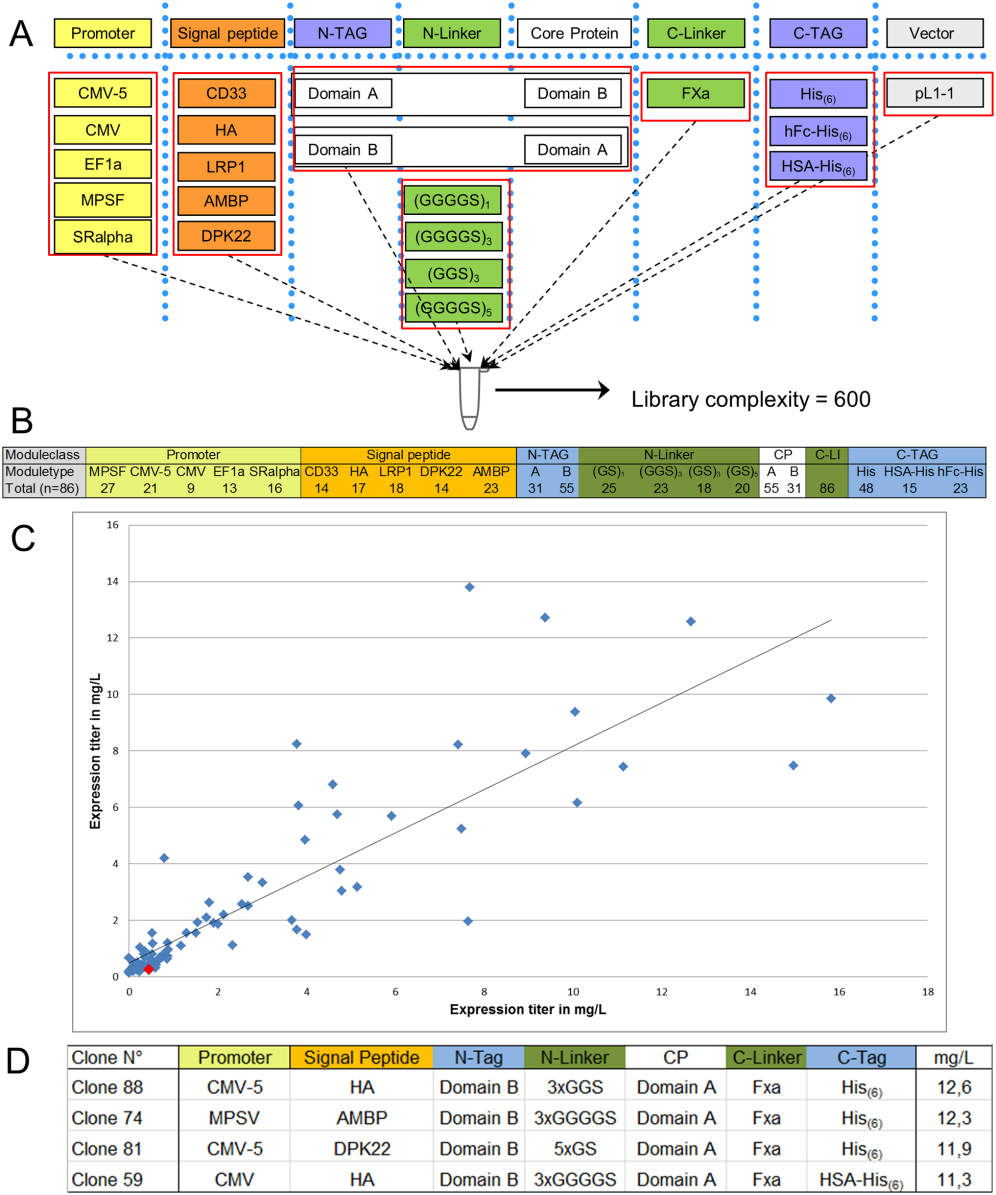
Expression optimization library of an artificial two domain cytokine. (A) Cytokine expression optimization library design. Listed functional modules were included in two separate reactions. In one reaction domain A is used at the N-TAG position and domain B at the core protein position. In the second reaction domain B is used at the N-TAG position and domain A at the core protein position. The resulting final library has a theoretical complexity of 600. (B) Distribution of the implemented modules in accordance to their functional class of a test set of 88 randomly selected clones. (C) Expression titer of the 88 randomly selected clones as determined by ELISA in two independent experiments (Pearson’s Correlation r = 0.8642, R2 = 0.7468, P (two-tailed) <0.0001). Red diamond represents the starting expression level. All clones are correctly assembled and include all functions essential for expression like promoter and signal peptide. (D) Construct design of the Top 4 expression constructs.

In this artificial protein, two single proteins were connected now as subunits by a linker sequence. We included five promoters, five signal peptides and used the N-TAG position for one of the two subunits. The two subunits are separated by four different linkers and C-terminally fused to three tags which support purification but can be removed by an integrated FXa protease cleavage site (Fig 4A). The theoretical complexity of this expression scouting and optimization library would be 1200. In order to avoid the generation of unproductive homodimers (AA and BB) two independent assembly reactions were performed. In the first assembly reaction domain A is used at the N-TAG position and domain B at the core protein position. In the second assembly reaction domain B is used at the N-TAG position and domain A at the core protein position. In both reactions the two domain modules where combined with the other selected 19 modules. The two libraries where combined prior transformation resulting in a total complexity of 600. From this library, 88 constructs were selected randomly and Sanger sequencing identified 86 unique expression clones. All modules used during library construction were identified in the final library and modules of the same type were found with acceptable frequencies, which are sufficient for high throughput screening (Fig 4B). For the C-terminal tags however, some greater bias towards the small His-tag was observed (P < 0.0001; Chi-square test). This observation may be based on the significant size difference between His-tag (18 nucleotides) and for example HSA-His tag (1779 nucleotides). DNA of the 86 randomly chosen expression constructs was prepared twice in independent DNA preparations, concentration adjusted, transfected in HEK-293-6E cells, cultivated for three days and expression in the cell supernatants was measured by a specific ELISA to domain A. The correlation plot of the two expression experiments is shown in Fig 4C, with the red diamond symbol representing the starting expression level. Like in the initial experiments the majority of constructs show a mean expression level of below 1 mg/L (47/86) in the range of the starting construct (0.3 mg/L). However the top constructs achieve an expression level of >12 mg/L. Analysis of the 4 top performing clones, all having an average expression level of > 10 mg/L, showed a diverse and unpredicted module composition. However, a strong tendency for the order: domain B—domain A and the preference of the short His-tag, was evident and also reflected by the whole set of 86 analyzed variants (Fig 4D). Using this approach an improvement of approximately 60-fold could be achieved already by a moderately sized expression optimization library.

## Discussion

The modular expression toolbox described herein provides a scientist with multiple options to very easily either engineer proteins or to generate libraries of expression constructs. As construct design specification and assembly processes are standardized and highly efficient, the scientists focus can be purely on the generation of the optimal protein design, without the burden of defining and adjusting the cloning and assembly strategy for each protein or even for multiple proteins in a given project. The envisioned modules are simply selected on the drawing board and combined in a single one-pot reaction. The described built-in design specifications, like definition of eight functional modules with unique standardized fusion sites that are validated for high assembly efficiency, enables a Lego^®^ brick-like assembly feeling. The flexibility can be further increased by understanding that the number of positions and their nominal assignment to a function only sets an initial frame for the system. There are multiple options to flexibly extend and adjust the system without losing the advantages. The example of the successful expression optimization approach for the artificial two domain cytokine demonstrated clearly, that module positions can be freely allocated to other functions, like the first domain of the POI using the position designated for N-term linker. Also, the number of modules is not fixed to eight and can be adapted to the specific needs of a given project. In cases where not all module positions are needed, skipped positions can be bridged by a modified POI module. Here like in the first example the POI would start with G-GCT and ends with GGTT. When additional module positions are needed, however, an existing module can be split into two new module positions separated by a newly defined, unique fusion site. By keeping the outer fusion sites of the original module the compatibility to MoPET is ensured at the same time.

The complete modularization of the MoPET system enables also the gradually expansion of the system with new functionalities like new promoters or linkers in a simple and resource efficient way by adding one additional level 0 module to the system. In comparison to vector systems in which multiple functional parts in different combinations are fixed in a given plasmid backbone, the expansion by one additional promoter for example, would require the generation and modification of multiple plasmids which is time consuming and resource intensive. Reduction of the vector backbone to the most essential functions, selection marker and replication origin, allows an easy adaptation of the MoPET system to further eukaryotic and even prokaryotic systems. This can be achieved by adding one backbone module with the appropriate selection markers and specific origins of replication for the anticipated expression system. Furthermore a set of organism specific promoter (eukaryotic) or promoter/RBS combinations (prokaryotic) would have to be generated. But even in the case of the expansion from a eukaryotic to a prokaryotic system, all linker and tag modules can be recycled between different projects. The pre-defined design specification combined with a highly efficient assembly process however makes it not only possible to generate efficiently expression constructs designed rationally by the user. Especially in cases where either no information about a suitable starting design is available, or the initial expression designs did not result in sufficient expression levels, the MoPET system and the included set of functional modules can be used in a library and screening mode. In contrast to library generation technologies based on DNA-oligos or PCR steps or combinations thereof, the MoPET system does not need the design of complex and time consuming case by case cloning strategies which are linked to the synthesis of defined sets of DNA oligos. This time and resource advantage is combined by a perfect control of module composition and the relative abundance of each module. In contrast to PCR-based technologies this tight control is also applicable for short DNA stretches coding for tags or linkers consisting only of 4 amino acids.

In contrast to the tight control and high predictability for the library assembly process itself, the interdependencies of multiple variables influencing generation of a suitable expression construct are not understood. The two analyzed libraries herein following the screening approach, showed a relationship between the number of analyzed constructs and the relative expression improvement. In the 1^st^ library 22 variants were needed to get a 2–3 fold improvement, whereas in the 2^nd^ library out of 88 variants a 60 fold improvement could be achieved. This would indicate that for higher improvements of relative expression, large numbers of variants have to be screened. One solution could be a stronger HTS support to further increase expression and screening throughput. However analysis of the 2^nd^ library revealed a further option. Based on the results a preferred construct design was emerging after the 1^st^ screening round, with one defined domain orientation and a His_(6)_-tag preferred at the C-terminus. Using the same module set further combinations could be explored using e.g. different plasmid backbones and combinations of further signal peptides to further increase the expression gain of the described preferred start configuration.

In contrast to the subunit orientation and preferred C-terminal linker, promoter and signal peptide combinations for the top variants did not show a clear picture, which could indicate that they are neutral in terms of the expression level of a given construct. Surprisingly however nearly identical constructs differing in e.g. just the signal peptide show dramatic drops in expression, highlighting that presumably only specific combinations of functional modules are giving optimal results.

## Supporting information

S1 Fig General principle of the Golden Gate assembly used in the MoPET system. A detailed overview of the organization and orientation of the type IIS restriction sites and the fusion sites at the different levels of the MoPET system is shown. Level 0 modules are flanked by BsaI recognition sites and module specific fusion sites (fs) are highlighted with color. Promoter (P) and Signal peptide (SP) are shown as example. The level 0 promoter module and the other level 0 modules required to form a complete expression construct (not shown) are then assembled via BsaI into a level 1 destination vector, creating the final level 1 expression construct.
(TIF)

S1 File Module sequence information. Fasta sequences of all coding module sequences.
(RTF)
