# Supplementary figures and images for: Modular Protein Expression Toolbox (MoPET), a standardized assembly system for defined expression constructs and expression optimization libraries
Source: PLoS One. 2017 May 17;12(5):e0176314. doi: 10.1371/journal.pone.0176314 (PMC5435135; doi:10.1371/journal.pone.0176314)

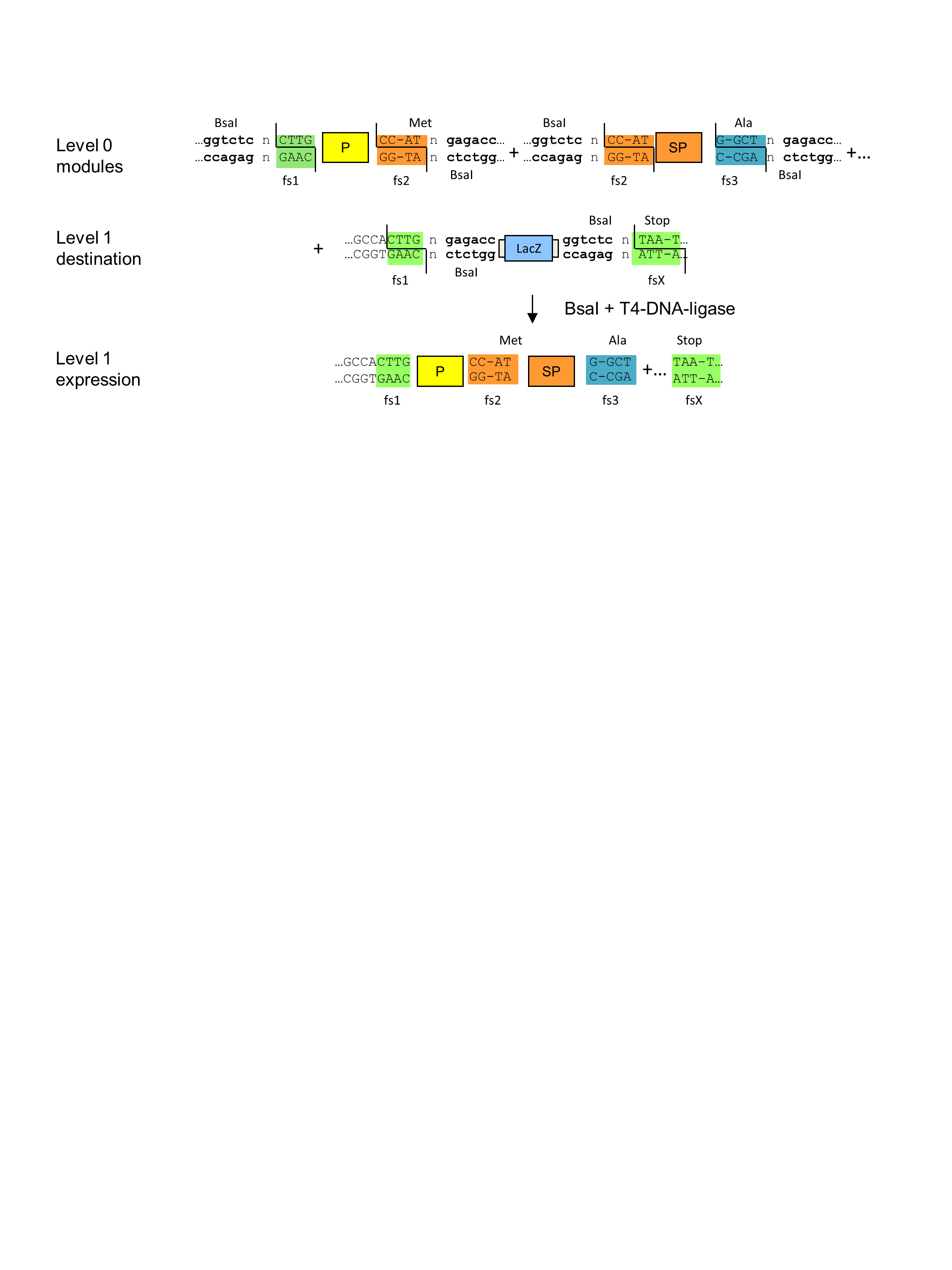

Supplement: S1 Fig — A detailed overview of the organization and orientation of the type IIS restriction sites and the fusion sites at the different levels of the MoPET system is shown. Level 0 modules are flanked by BsaI recognition sites and module specific fusion sites (fs) are highlighted with color. Promoter (P) and Signal peptide (SP) are shown as example. The level 0 promoter module and the other level 0 modules required to form a complete expression construct (not shown) are then assembled via BsaI into a level 1 destination vector, creating the final level 1 expression construct. (TIF) [file pone.0176314.s001.tif]
