# Supplementary material for: Modular Protein Expression Toolbox (MoPET), a standardized assembly system for defined expression constructs and expression optimization libraries
Source: PLoS One. 2017 May 17;12(5):e0176314. doi: 10.1371/journal.pone.0176314 (PMC5435135; doi:10.1371/journal.pone.0176314)
Supplement: S1 File — Fasta sequences of all coding module sequences. (RTF) [file pone.0176314.s002.rtf]

>Signal peptide CD33
GGTCTCTCCATGCCGCTGCTGCTACTGCTGCCCCTGCTGTGGGCAGGGGCCCTGGCTAGAGACC

>Signal peptide IGHV4-30-2
GGTCTCTCCATGAAACACCTGTGGTTTTTTCTGCTGCTGGTTGCAGCACCGCGTTGGGTTCTGGCTAGAGACC

>Signal peptide IGLV2-8
GGTCTCTCCATGGCATGGGCACTGCTGCTGCTGACCCTGCTGACACAGGGCACCGGTAGCTGGGCTAGAGACC

>Signal peptide DPK22
GGTCTCTCCATGGAAACCCCAGCGCAGCTTCTCTTCCTCCTGCTACTCTGGCTCCCAGATACCACGGCTAGAGACC

>Signal peptide IGKV4-1
GGTCTCTCCATGGTGTTGCAGACCCAGGTGTTCATTTCTCTGTTGCTCTGGATCTCTGGTGCCCTGGCTAGAGACC

>Signal peptide HA
GGTCTCTCCATGGCCATCATCTACCTGATCCTGCTGTTCACCGCCGTGCGGAGGGCTAGAGACC

>Signal peptide kappa
GGTCTCTCCATGGAGACAGACACACTCCTGCTATGGGTACTGCTGCTCTGGGTTCCAGGTTCCACGGCTAGAGACC

>Signal peptide LRP1
GGTCTCTCCATGCTGACCCCCCCACTGCTGCTGCTGCTGCCTCTGCTGTCTGCCCTGGTGGCGGCTAGAGACC

>Signal peptide AMBP+50
GGTCTCTCCATGAGGAGCCTCGGGGCCCTGCTCTTGCTGCTGAGCGCCTGCCTGGCGGTGAGCGCTGGCCCTGTGCCAACGCCGCCCGACAACATCCAAGTGCAGGAAAACTTCAATATCTCTCGGATCTATGGGAAGTGGTACAACCTGGCCATCGGTTCCACCTGCCCCTGGCTGAAGAAGATCATGGCTAGAGACC

>Signal peptide AMBP
GGTCTCTCCATGAGGAGCCTCGGGGCCCTGCTCTTGCTGCTGAGCGCCTGCCTGGCGGTGTCGGCTAGAGACC

>N-TAG His(6)
GGTCTCTGGCTCATCATCATCACCATCATGGATAGAGACC

>N-TAG His(6)-myc
GGTCTCTGGCTCATCATCATCACCATCATGAACAGAAACTGATTTCTGAAGAGGATCTGGGATAGAGACC

>N-TAG Avi-tag
GGTCTCTGGCTTCCGGCCTGAACGACATCTTCGAGGCTCAGAAAATCGAATGGCACGAAGGATAGAGACC

>N-TAG His(6)- HSA
GGTCTCTGGCTCATCATCATCACCATCATGGATCCGATGCCCACAAGTCTGAGGTGGCCCACCGGTTCAAGGACCTGGGCGAGGAAAACTTCAAGGCCCTGGTGCTGATCGCCTTCGCCCAGTACCTGCAGCAGTGCCCCTTCGAGGACCACGTGAAGCTGGTCAACGAAGTGACCGAGTTCGCCAAGACCTGCGTGGCCGACGAGAGCGCCGAGAACTGCGACAAGAGCCTGCACACCCTGTTCGGCGACAAGCTGTGCACCGTGGCCACCCTGCGGGAAACCTACGGCGAGATGGCCGACTGCTGCGCCAAGCAGGAACCCGAGCGGAACGAGTGCTTCCTGCAGCACAAGGACGACAACCCCAACCTGCCCAGACTCGTGCGGCCTGAGGTGGACGTGATGTGCACCGCCTTCCACGACAACGAGGAAACCTTCCTGAAGAAGTACCTGTACGAGATCGCCAGACGGCACCCCTACTTCTACGCCCCCGAGCTGCTGTTCTTCGCCAAGCGGTACAAGGCCGCCTTCACCGAGTGTTGCCAGGCCGCCGATAAGGCCGCCTGCCTGCTGCCTAAGCTGGACGAGCTGCGGGATGAGGGCAAGGCCAGCTCCGCCAAGCAGAGACTGAAGTGCGCCAGCCTGCAGAAGTTCGGCGAGCGGGCCTTTAAGGCCTGGGCCGTGGCCAGACTGAGCCAGAGATTCCCCAAGGCCGAGTTTGCCGAGGTGTCCAAGCTGGTCACCGACCTGACCAAGGTGCACACCGAGTGCTGTCACGGCGACCTGCTGGAATGCGCCGACGACAGAGCCGATCTGGCCAAGTACATCTGCGAGAACCAGGACAGCATCAGCAGCAAGCTGAAAGAGTGCTGCGAGAAGCCCCTGCTGGAAAAGAGCCACTGTATCGCCGAGGTGGAGAACGACGAGATGCCCGCCGATCTGCCTAGCCTGGCCGCCGACTTCGTGGAGAGCAAGGACGTGTGCAAGAACTACGCCGAGGCCAAGGATGTGTTCCTGGGCATGTTCCTGTACGAGTACGCCCGCAGACACCCCGATTACAGCGTGGTGCTGCTGCTGCGGCTGGCCAAGACCTACGAGACAACCCTGGAAAAGTGCTGCGCCGCTGCCGATCCTCACGAGTGCTACGCCAAGGTGTTCGACGAGTTCAAGCCTCTGGTGGAGGAACCCCAGAACCTGATCAAGCAGAACTGCGAGCTGTTCGAGCAGCTGGGCGAGTACAAGTTCCAGAACGCCCTGCTCGTGCGGTACACCAAGAAAGTGCCCCAGGTGTCCACCCCTACCCTGGTGGAGGTGTCCCGGAACCTGGGCAAAGTGGGCAGCAAGTGCTGCAAGCACCCTGAGGCCAAGAGAATGCCCTGCGCCGAGGACTACCTGTCCGTGGTGCTGAATCAGCTGTGCGTGCTGCACGAGAAAACCCCCGTGTCCGACAGAGTGACCAAGTGCTGTACCGAGAGCCTGGTCAACAGACGGCCCTGCTTCAGCGCCCTGGAAGTGGACGAGACATACGTGCCCAAAGAGTTCAACGCCGAGACATTCACCTTCCACGCCGACATCTGCACCCTGAGCGAGAAAGAGCGGCAGATCAAGAAACAGACCGCCCTGGTGGAACTGGTCAAGCACAAGCCCAAGGCCACCAAAGAACAGCTGAAGGCCGTGATGGACGACTTCGCCGCCTTTGTGGAGAAATGCTGCAAGGCCGACGACAAAGAGACATGCTTCGCCGAGGAAGGCAAGAAGCTGGTCGCCGCCTCCCAGGCAGCTCTGGGACTGGGATAGAGACC

>N-TAG His(6)- hIgG1-Fc
GGTCTCTGGCTCACCACCATCACCATCACGACAAGACCCACACCTGTCCCCCTTGTCCTGCCCCTGAACTGCTGGGCGGACCTAGCGTGTTCCTGTTCCCCCCAAAGCCCAAGGACACCCTGATGATCTCCCGGACCCCCGAAGTGACCTGCGTGGTGGTGGATGTGTCCCACGAGGACCCTGAAGTGAAGTTTAATTGGTACGTGGACGGCGTGGAAGTGCACAACGCCAAGACCAAGCCCAGAGAGGAACAGTACAACAGCACCTACCGGGTGGTGTCCGTGCTGACCGTGCTGCACCAGGACTGGCTGAATGGGAAAGAGTACAAGTGCAAAGTGTCCAACAAGGCCCTGCCTGCCCCCATCGAGAAAACCATCAGCAAGGCCAAGGGCCAGCCCCGCGAACCCCAGGTGTACACACTGCCCCCTAGCAGGGACGAGCTGACCAAGAACCAGGTGTCCCTGACCTGTCTCGTGAAGGGCTTCTACCCCTCCGATATCGCCGTGGAATGGGAGAGCAACGGCCAGCCTGAGAACAACTACAAGACCACCCCCCCTGTGCTGGACTCCGACGGCTCATTCTTCCTGTACAGCAAACTGACCGTGGATAAGAGCCGGTGGCAGCAGGGCAACGTGTTCAGCTGCAGCGTGATGCACGAGGCCCTGCACAACCACTACACCCAGAAGTCCCTGAGCCTGAGCCCCGGCAAGGGATAGAGACC

>N-TAG hIgG1-Fc
GGTCTCTGGCTGACAAGACCCACACCTGTCCCCCTTGTCCTGCCCCTGAACTGCTGGGCGGACCTAGCGTGTTCCTGTTCCCCCCAAAGCCCAAGGACACCCTGATGATCTCCCGGACCCCCGAAGTGACCTGCGTGGTGGTGGATGTGTCCCACGAGGACCCTGAAGTGAAGTTTAATTGGTACGTGGACGGCGTGGAAGTGCACAACGCCAAGACCAAGCCCAGAGAGGAACAGTACAACAGCACCTACCGGGTGGTGTCCGTGCTGACCGTGCTGCACCAGGACTGGCTGAATGGGAAAGAGTACAAGTGCAAAGTGTCCAACAAGGCCCTGCCTGCCCCCATCGAGAAAACCATCAGCAAGGCCAAGGGCCAGCCCCGCGAACCCCAGGTGTACACACTGCCCCCTAGCAGGGACGAGCTGACCAAGAACCAGGTGTCCCTGACCTGTCTCGTGAAGGGCTTCTACCCCTCCGATATCGCCGTGGAATGGGAGAGCAACGGCCAGCCTGAGAACAACTACAAGACCACCCCCCCTGTGCTGGACTCCGACGGCTCATTCTTCCTGTACAGCAAACTGACCGTGGATAAGAGCCGGTGGCAGCAGGGCAACGTGTTCAGCTGCAGCGTGATGCACGAGGCCCTGCACAACCACTACACCCAGAAGTCCCTGAGCCTGAGCCCCGGCAAGGGATAGAGACC

>N-TAG FLAG
GGTCTCTGGCTGACTACAAGGACGACGACGACAAGGGATAGAGACC

>N-TAG StrepII
GGTCTCTGGCTTGGAGCCACCCCCAGTTCGAGAAGGGATAGAGACC

>N-Linker (GGGGS)1
GGTCTCTGGATCCGGTGGTGGTGGTTCCGGGAGAGACC

>N-Linker (GGGGS)3
GGTCTCTGGATCCGGCGGTGGTGGTAGTGGTGGCGGTGGTTCAGGCGGTGGCGGCTCCGGGAGAGACC

>N-Linker (GGS)3
GGTCTCTGGATCCGGCGGAAGCGGAGGCAGCGGGGGATCCGGGAGAGACC

>N-Linker (GGGGS)5
GGTCTCTGGATCCGGCGGAGGCGGATCTGGCGGCGGAGGAAGTGGCGGAGGGGGCTCTGGGGGAGGCGGCAGTGGCGGGGGAGGATCCGGGAGAGACC

>N-Linker TEV
GGTCTCTGGATCCGAAAACCTGTATTTTCAGGGCGGGAGAGACC

>N-Linker FXa
GGTCTCTGGATCCATCGAGGGCCGCATGGACGGGAGAGACC

>C-Linker (GGGGS)1
GGTCTCTGGTGGTGGTGGTGGTTCAGGCAGAGACC

>C-Linker (GGGGS)3
GGTCTCTGGTGGCGGTGGTAGTGGTGGCGGTGGTTCAGGCGGTGGCGGCTCAGGCAGAGACC

>C-Linker (GGS)3
GGTCTCTGGTGGAAGCGGAGGCAGCGGGGGATCAGGCAGAGACC

>C-Linker (GGGGS)5
GGTCTCTGGTGGCGGAGGATCTGGCGGAGGCGGAAGTGGCGGCGGAGGAAGCGGAGGGGGCGGATCAGGCGGGGGAGGATCAGGCAGAGACC

>C-Linker TEV
GGTCTCTGGTGAAAACCTGTATTTTCAAGGCAGAGACC

>C-Linker (GGGGS)7
GGTCTCTGGTGGCGGAGGATCTGGCGGAGGCGGAAGTGGCGGCGGAGGAAGCGGAGGGGGCGGATCAGGCGGGGGAGGCAGTGGGGGAGGGGGATCAGGGGGAGGCGGCTCAGGCAGAGACC

>C-Linker  FXa
GGTCTCTGGTGGCATCGAGGGCCGCATGGATGGAGGCAGAGACC

> C-Linker (GGGGS)9
GGTCTCTGGTGGCGGAGGATCTGGCGGAGGCGGAAGTGGCGGCGGAGGAAGCGGAGGGGGCGGATCAGGCGGGGGAGGCAGTGGGGGAGGGGGATCAGGGGGAGGCGGCTCTGGGGGGGGAGGCAGCGGAGGCGGAGGATCAGGCAGAGACC

> C-Linker (GGGGS)11
GGTCTCTGGTGGCGGAGGATCTGGCGGAGGCGGAAGTGGCGGCGGAGGAAGCGGAGGGGGCGGATCAGGCGGGGGAGGCAGTGGGGGAGGGGGATCAGGGGGAGGCGGCTCTGGGGGGGGAGGCAGCGGAGGCGGAGGCTCAGGGGGAGGGGGGAGCGGCGGAGGGGGATCAGGCAGAGACC

>C-Linker (GGGGS)13
GGTCTCTGGTGGCGGAGGATCTGGCGGAGGCGGAAGTGGCGGCGGAGGAAGCGGAGGGGGCGGATCAGGCGGGGGAGGCAGTGGGGGAGGGGGATCAGGGGGAGGCGGCTCTGGGGGGGGAGGCAGCGGAGGCGGAGGCTCAGGGGGAGGGGGGAGCGGCGGAGGGGGCAGCGGAGGGGGAGGAAGTGGCGGAGGCGGCTCAGGCAGAGACC

>C-Linker (GGGGS)15
GGTCTCTGGTGGCGGAGGATCTGGCGGAGGCGGAAGTGGCGGCGGAGGAAGCGGAGGGGGCGGATCAGGCGGGGGAGGCAGTGGGGGAGGGGGATCAGGGGGAGGCGGCTCTGGGGGGGGAGGCAGCGGAGGCGGAGGCTCAGGGGGAGGGGGGAGCGGCGGAGGGGGCAGCGGAGGGGGAGGAAGTGGCGGAGGCGGCTCCGGCGGAGGGGGATCTGGCGGGGGAGGATCAGGCAGAGACC

>C-TAG c-tag
GGTCTCTAGGCGAACCCGAAGCCTAATAGAGACC

>C-TAG Avi-tag
GGTCTCTAGGCTCCGGCCTGAACGACATCTTCGAGGCTCAGAAAATCGAATGGCACGAAGGCTAATAGAGACC

> C-TAG His(6)
GGTCTCTAGGCCATCATCATCACCATCATTAATAGAGACC

>C-TAG  DKTH hFc-His(6)
GGTCTCTAGGCGACAAGACCCACACCTGTCCCCCTTGTCCTGCCCCTGAACTGCTGGGCGGACCTAGCGTGTTCCTGTTCCCCCCAAAGCCCAAGGACACCCTGATGATCTCCCGGACCCCCGAAGTGACCTGCGTGGTGGTGGATGTGTCCCACGAGGACCCTGAAGTGAAGTTTAATTGGTACGTGGACGGCGTGGAAGTGCACAACGCCAAGACCAAGCCCAGAGAGGAACAGTACAACAGCACCTACCGGGTGGTGTCCGTGCTGACCGTGCTGCACCAGGACTGGCTGAATGGGAAAGAGTACAAGTGCAAAGTGTCCAACAAGGCCCTGCCTGCCCCCATCGAGAAAACCATCAGCAAGGCCAAGGGCCAGCCCCGCGAACCCCAGGTGTACACACTGCCCCCTAGCAGGGACGAGCTGACCAAGAACCAGGTGTCCCTGACCTGTCTCGTGAAGGGCTTCTACCCCTCCGATATCGCCGTGGAATGGGAGAGCAACGGCCAGCCTGAGAACAACTACAAGACCACCCCCCCTGTGCTGGACTCCGACGGCTCATTCTTCCTGTACAGCAAACTGACCGTGGATAAGAGCCGGTGGCAGCAGGGCAACGTGTTCAGCTGCAGCGTGATGCACGAGGCCCTGCACAACCACTACACCCAGAAGTCCCTGAGCCTGAGCCCCGGCAAGCACCACCATCACCATCACTAATAGAGACC

>C-TAG  PKSC hFc-His(6)
GGTCTCTAGGCCCTAAGAGCTGCGACAAGACCCACACCTGTCCCCCTTGTCCTGCCCCTGAACTGCTGGGCGGACCTAGCGTGTTCCTGTTCCCCCCAAAGCCCAAGGACACCCTGATGATCTCCCGGACCCCCGAAGTGACCTGCGTGGTGGTGGATGTGTCCCACGAGGACCCTGAAGTGAAGTTTAATTGGTACGTGGACGGCGTGGAAGTGCACAACGCCAAGACCAAGCCCAGAGAGGAACAGTACAACAGCACCTACCGGGTGGTGTCCGTGCTGACCGTGCTGCACCAGGACTGGCTGAATGGGAAAGAGTACAAGTGCAAAGTGTCCAACAAGGCCCTGCCTGCCCCCATCGAGAAAACCATCAGCAAGGCCAAGGGCCAGCCCCGCGAACCCCAGGTGTACACACTGCCCCCTAGCAGGGACGAGCTGACCAAGAACCAGGTGTCCCTGACCTGTCTCGTGAAGGGCTTCTACCCCTCCGATATCGCCGTGGAATGGGAGAGCAACGGCCAGCCTGAGAACAACTACAAGACCACCCCCCCTGTGCTGGACTCCGACGGCTCATTCTTCCTGTACAGCAAACTGACCGTGGATAAGAGCCGGTGGCAGCAGGGCAACGTGTTCAGCTGCAGCGTGATGCACGAGGCCCTGCACAACCACTACACCCAGAAGTCCCTGAGCCTGAGCCCCGGCAAGCACCACCATCACCATCACTAATAGAGACC

>C-TAG  HSA-His(6)
GGTCTCTAGGCGATGCCCACAAGTCTGAGGTGGCCCACCGGTTCAAGGACCTGGGCGAGGAAAACTTCAAGGCCCTGGTGCTGATCGCCTTCGCCCAGTACCTGCAGCAGTGCCCCTTCGAGGACCACGTGAAGCTGGTCAACGAAGTGACCGAGTTCGCCAAGACCTGCGTGGCCGACGAGAGCGCCGAGAACTGCGACAAGAGCCTGCACACCCTGTTCGGCGACAAGCTGTGCACCGTGGCCACCCTGCGGGAAACCTACGGCGAGATGGCCGACTGCTGCGCCAAGCAGGAACCCGAGCGGAACGAGTGCTTCCTGCAGCACAAGGACGACAACCCCAACCTGCCCAGACTCGTGCGGCCTGAGGTGGACGTGATGTGCACCGCCTTCCACGACAACGAGGAAACCTTCCTGAAGAAGTACCTGTACGAGATCGCCAGACGGCACCCCTACTTCTACGCCCCCGAGCTGCTGTTCTTCGCCAAGCGGTACAAGGCCGCCTTCACCGAGTGTTGCCAGGCCGCCGATAAGGCCGCCTGCCTGCTGCCTAAGCTGGACGAGCTGCGGGATGAGGGCAAGGCCAGCTCCGCCAAGCAGAGACTGAAGTGCGCCAGCCTGCAGAAGTTCGGCGAGCGGGCCTTTAAGGCCTGGGCCGTGGCCAGACTGAGCCAGAGATTCCCCAAGGCCGAGTTTGCCGAGGTGTCCAAGCTGGTCACCGACCTGACCAAGGTGCACACCGAGTGCTGTCACGGCGACCTGCTGGAATGCGCCGACGACAGAGCCGATCTGGCCAAGTACATCTGCGAGAACCAGGACAGCATCAGCAGCAAGCTGAAAGAGTGCTGCGAGAAGCCCCTGCTGGAAAAGAGCCACTGTATCGCCGAGGTGGAGAACGACGAGATGCCCGCCGATCTGCCTAGCCTGGCCGCCGACTTCGTGGAGAGCAAGGACGTGTGCAAGAACTACGCCGAGGCCAAGGATGTGTTCCTGGGCATGTTCCTGTACGAGTACGCCCGCAGACACCCCGATTACAGCGTGGTGCTGCTGCTGCGGCTGGCCAAGACCTACGAGACAACCCTGGAAAAGTGCTGCGCCGCTGCCGATCCTCACGAGTGCTACGCCAAGGTGTTCGACGAGTTCAAGCCTCTGGTGGAGGAACCCCAGAACCTGATCAAGCAGAACTGCGAGCTGTTCGAGCAGCTGGGCGAGTACAAGTTCCAGAACGCCCTGCTCGTGCGGTACACCAAGAAAGTGCCCCAGGTGTCCACCCCTACCCTGGTGGAGGTGTCCCGGAACCTGGGCAAAGTGGGCAGCAAGTGCTGCAAGCACCCTGAGGCCAAGAGAATGCCCTGCGCCGAGGACTACCTGTCCGTGGTGCTGAATCAGCTGTGCGTGCTGCACGAGAAAACCCCCGTGTCCGACAGAGTGACCAAGTGCTGTACCGAGAGCCTGGTCAACAGACGGCCCTGCTTCAGCGCCCTGGAAGTGGACGAGACATACGTGCCCAAAGAGTTCAACGCCGAGACATTCACCTTCCACGCCGACATCTGCACCCTGAGCGAGAAAGAGCGGCAGATCAAGAAACAGACCGCCCTGGTGGAACTGGTCAAGCACAAGCCCAAGGCCACCAAAGAACAGCTGAAGGCCGTGATGGACGACTTCGCCGCCTTTGTGGAGAAATGCTGCAAGGCCGACGACAAAGAGACATGCTTCGCCGAGGAAGGCAAGAAGCTGGTCGCCGCCTCCCAGGCAGCTCTGGGACTGCACCATCATCACCATCACTAATAGAGACC

>C-TAG  StrepII-His(6)
GGTCTCTAGGCTGGTCCCACCCCCAGTTCGAGAAGCACCACCACCATCACCACTAATAGAGACC

>C-TAG  FLAG- His(6)
GGTCTCTAGGCGACTACAAGGACGACGACGACAAGCACCACCACCATCACCACTAATAGAGACC

>C-TAG  Myc- His(6)
GGTCTCTAGGCGAGCAGAAGCTGATCTCCGAAGAGGACCTGCACCACCACCATCACCACTAATAGAGACC

>C-TAG  mFc- His(6)
GGTCTCTAGGCGTGCCCAGAGACTGCGGCTGCAAGCCTTGCATCTGCACCGTGCCTGAGGTGTCCAGCGTGTTCATCTTCCCACCCAAGCCCAAGGACGTGCTGACCATCACCCTGACCCCCAAAGTGACCTGCGTGGTGGTGGACATCAGCAAGGACGACCCCGAGGTGCAGTTCAGTTGGTTCGTGGACGACGTGGAAGTGCACACCGCCCAGACCCAGCCCAGAGAGGAACAGTTCAACAGCACCTTCAGAAGCGTGTCCGAGCTGCCCATCATGCACCAGGACTGGCTGAACGGCAAAGAATTCAAGTGCAGAGTGAACAGCGCCGCCTTCCCTGCCCCCATCGAGAAAACCATCTCCAAGACCAAGGGCAGACCCAAGGCCCCCCAGGTGTACACAATCCCCCCACCCAAAGAACAGATGGCCAAGGACAAGGTGTCCCTGACCTGCATGATCACCGATTTCTTCCCAGAGGACATCACCGTGGAATGGCAGTGGAACGGCCAGCCCGCCGAGAACTACAAGAACACCCAGCCTATCATGGACACCGACGGCAGCTACTTCGTGTACAGCAAGCTGAACGTGCAGAAGTCCAACTGGGAGGCCGGCAACACCTTCACCTGTAGCGTGCTGCACGAGGGCCTGCACAACCACCACACCGAGAAGTCCCTGAGCCACAGCCCCGGCAAGCACCACCATCACCATCACTAATAGAGACC
